# Cyberattack Models for Ship Equipment Based on the MITRE ATT&CK Framework

**DOI:** 10.3390/s22051860

**Published:** 2022-02-26

**Authors:** Yonghyun Jo, Oongjae Choi, Jiwoon You, Youngkyun Cha, Dong Hoon Lee

**Affiliations:** 1 DSLAB Company Ltd., Seoul 08511, Korea; yhjo@dslabcompany.com (Y.J.); wj.choi@dslabcompany.com (O.C.); jw.yoo@dslabcompany.com (J.Y.); 2 Graduate School of Information Security, Korea University, 145 Anam-ro, Seongbuk-gu, Seoul 02841, Korea; ykcha@korea.ac.kr

**Keywords:** maritime cybersecurity, cyber threat, MITRE ATT & CK, information sharing, security risk analysis

## Abstract

Cybersecurity is important on ships that use information and communication technology. On such ships, the work, control, and sensor systems are connected for steering, navigation, and cargo management inside the hull, and a cyberattack can have physical consequences such as sinking and crashing. Research on ship cybersecurity is a new challenge, and related studies are lacking. Cyberattack models can provide better insight. With this study, we aim to introduce a cyberattack analysis method based on the MITRE ATT&CK framework so that a cyberattack model for ships can be established. In addition, we identify the characteristics of the attack phase by analyzing cases of hacking and vulnerability research for ship systems using tactics, techniques, and procedures, and suggest the minimum measures essential for defense. Using the ship cyberattack model, we aim to identify the characteristics of the systems used for ship navigation, communication, and control; provide an understanding of the threats and vulnerabilities; and suggest mitigation measures through the proposed model. We believe the results of this study could guide future research.

## 1. Introduction

Modern ships are equipped with ship navigation and automation systems so that fuel can be saved by selecting efficient routes while complying with greenhouse gas emission regulations. They are also equipped with onboard Internet of Things (IoT) so that data can be exchanged with land in real time [1]. The convergence of information and communication technology on ships has led to new changes in ship design, construction, and safety technologies. The shipbuilding industry monitors the operation information of ship engines, controllers, and various navigation equipment in real time from land-based control centers through marine satellite communication. A smart ship is a ship that can be remotely diagnosed and controlled by an integrated onboard system. The International Maritime Organization (IMO) defines autonomous ships as maritime autonomous surface ships (MASSs), which are based on smart ships. The navigation and operation of MASSs, from berthing to unberthing, are controlled by the system. A report by Inmarsat stated that 66% of ships collect data through onboard sensors, and that the use of marine satellite communication by ships will increase to 53% by 2025 [2]. As the number of contact points with the ship information and communication technology environment increases, the ship’s control system environment becomes more vulnerable to cyber threats. Cyberattacks on maritime officials have increased by 900%, from 50 cases in 2017 to 120 cases in 2018, 310 cases in 2019, and over 500 cases in 2020 [3]. Recent security vulnerability analyses and studies on the hacking of real ship systems have demonstrated that ships are also vulnerable to cyberattacks. In 2014, Santamarta demonstrated the vulnerabilities of several devices manufactured by major companies used as marine satellite communication equipment [4]. In 2014, Santamarta also discovered a vulnerability that could enable data in the voyage data recorder (VDR), which acts as a black box on a ship, to be tampered with remotely. In 2017, a security company researcher explained that a defect in the web application of CommBox, a Norwegian maritime satellite communication terminal produced by KVH Industries, could leak crew and ship information (such as the Automatic Identification System (AIS) information), which could lead to spear phishing attacks based on the personal information of the crew. In 2019, the United States Coast Guard (USCG) issued a marine alert after confirming that the onboard network of a container ship sailing into New York Port was infected with malware and unable to operate [5,6]. In 2019, a security vulnerability assessment of the Electronic Chart Display and Information System (ECDIS; version JAN-901B) mounted on a Japanese training ship discovered vulnerabilities in the server message block (SMB) used by Windows Embedded, an outdated operating system, which allowed remote attacks [7]. An investigation and quantification of the attack surfaces of ships in 2019 found that the email, NMEA 0183 protocol, ECDIS, AIS, Ship Planning System, Cobham Satcom terminal, and VDR used by crews were the attack surfaces posing the highest risk [8]. In 2021, the risk level of the network protocols NMEA 2000, NMEA 0183, and AIS used in the steering and navigation systems of ships was described to be higher than that of general TCP/IPv6 with respect to attack types such as denial-of-service (DoS) attacks, spoofing attacks, packet sniffing attacks, and relay or man-in-the-middle (MiTM) attacks [9]. In 2021, the Islamic Revolutionary Guard Corps (IRGC) investigated how to sink ships through a cyberattack on the ballast water management system (BWMS) [10].

Since the 2021 incident, maritime-related organizations such as shipbuilders, ship owners, the IMO, and classification societies in various countries have announced laws and regulations regarding cybersecurity on ships. The IMO released the Maritime Cyber Risk Management in Safety Management Systems to manage maritime cybersecurity from 1 January 2021. The Classification Society has also introduced a type of approval process for cybersecurity frameworks. A key requirement of the regulatory and type approvals related to maritime cybersecurity is the implementation of a systematic cyber risk management process for protecting core ship systems, such as bridge systems, cargo handling and management systems, and power control systems, by referring to ISO27001 or the NIST Cybersecurity Framework [11]. In this process, threats and security vulnerabilities are assessed and identified to develop detection and protection measures to reduce the likelihood that vulnerabilities are exploited. Furthermore, a plan is established to respond to cyber risks and the procedures for maintaining continuity during cybersecurity incidents are developed.

Identifying threats and vulnerabilities is an essential step in managing ship cybersecurity risks [12]. One ship consists of 700 to 900 units of propulsion control, steering, navigation, and communication equipment, which are provided by 80 to 100 manufacturers. Therefore, it is important to identify the cyber threats and vulnerabilities of equipment supplied by manufacturers and installed on ships. In this sense, a ship cybersecurity vulnerability/threat sharing platform would be an effective way for multiple stakeholders related to ships to update their information on cyber threats and security vulnerabilities associated with ship equipment and supply chains.

Therefore, with this study, we aim to gain insight by explaining the ship hacking model based on the MITER ATT&CK framework, based on the known ship security research and security vulnerabilities.

In this study, 15 major studies on ship hacking are analyzed using the MITRE ATT&CK framework. According to the findings of the analysis, common security measures to be considered in modern ships are identified, and a new matrix of security mitigation measures for the ship environment is proposed.

The remainder of this paper is structured as follows. Section 2 provides background on hacking case analysis methodology. Section 3 analyzes the four cases in detail and summarizes the analysis results. Section 4 draws the conclusions.

## 2. Background

### MITRE ATT&CK Framework

CKC follows a clearly defined linear step sequence, which is useful for understanding the cyberattack process. However, this model is not effective for analyzing the individual behaviors of an adversary, determining correlations between the detailed actions and tactical objectives of an adversary, or identifying the associations between data sources for attack and countermeasures for defense [13]. MITRE ATT&CK, unlike the Cyber Kill Chain, maps and indexes virtually everything regarding an intrusion from both the attack and defense sides. Therefore, MITRE ATT&CK is suitable for modeling various cyberattacks.

Yoon et al. [14] compare the attack on U.S. broadcasters in Iran and the cyberattack on U.S. lawmakers by Russia using the CKC model and MITRE ATT&CK and found the following.

Clearly classify recent cyberattacks into seven phases (① Reconnaissance; ② Weaponization; ③ Delivery; ④ Exploitation; ⑤ Installation; ⑥ Command and Control; ⑦ Action on Objectives);

- Express cyberattack technology correctly; and
- Suggest specific security measures.

**Figure 1:**
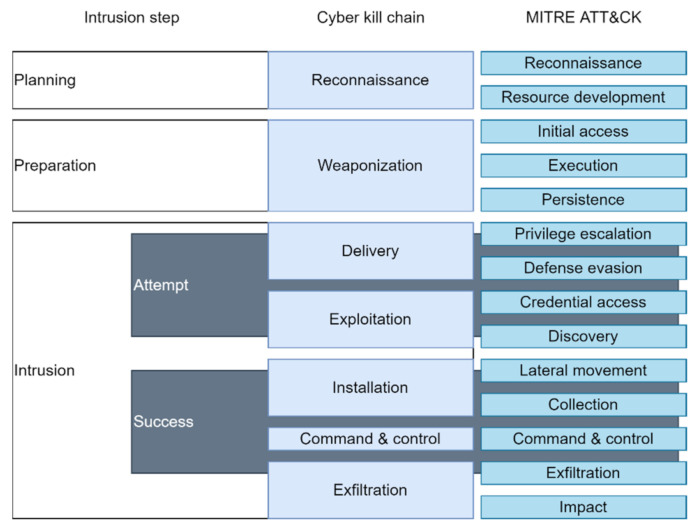
CKC vs. MITRE ATT&CK.

The MITRE ATT&CK framework documents and categorizes cyber adversary behavior into tactics, techniques, and procedures (TTPs) [15], which are not restricted by an order. Figure 1 compares the intrusion steps of CKC and MITRE ATT&CK.

MITRE ATT&CK is a security threat knowledge database that analyzes how an adversary invades and spreads through computer systems, focusing on data identified in actual breaches. Data are described by the three elements of TTP, which enables the construction of defense in depth (DiD).

- Tactics: the methods and objectives used in the attack;
- Techniques: 185 techniques and 367 sub-techniques have been identified as technical cyberattack methods for achieving tactical objectives;
- Procedures: the sequential processes of an attack.

MITRE updated the MITRE ATT&CK Enterprise framework in October 2020 by integrating *Reconnaissance* and *Resource Development* into two tactics for MITRE PRE-ATT&CK, which describes the preparation phase of an attack [16]. Table 1 describes each tactic in MITRE ATT&CK.

## 3. MITRE ATT&CK-Based Ship Cyberattack Analysis

Since IOActive [17] researchers conducted a vulnerability assessment study on ship black box systems in 2014, various types of vulnerability analyses and studies on the hacking of the communication, navigation, and steering management systems on ships have been published. This section analyzes ship hacking techniques reported in academia and by the maritime industry. Using MITRE ATT&CK analysis, the attack process and attack characteristics of each hacking technique are analyzed and organized using a consistent framework.

Santamarta [4] described the backdoors, hardcoded credentials, insecure protocols, undocumented protocols, and password reset weaknesses found in satellite communication terminals, further describing the falsification scenarios for ECDIS, ship status information, and cargo information based on the security vulnerabilities associated with SAILOR 900 VSAT (Cobham, Denmark) and JUE-250 FB (Japan Radio Co. Ltd., Tokyo, Japan) terminals.

Various cyberattack threats to ship AIS systems were classified by Kessler et al. [16], who argued that AIS malfunction, AIS jamming, and spoofing were the most commonly found attack types.

In addition, Lund et al. [18] described a cyberattack scenario arising from integrated navigation systems (INSs), electronic chart display, and information systems. In this scenario, the malware modifies the Global Positioning System (GPS) coordinates with an MiTM attack. The attack causes the ship’s system to crash to a “blue screen”, or the ship sails to different coordinates.

Svilicic et al. [7] performed a quantitative cyber risk assessment using a security vulnerability scanning tool on the JAN-901B ECDIS system (Japan Radio Co. Ltd., Tokyo, Japan), which was installed on the Japanese training ship *Fukae-maru*. The interface of this system consisted of an Ethernet local area network (LAN; 10/100 Mbps) and IEC61162-1/2, USB, and was run on a Windows XP system. Ten threats were found as a result.

Svilicic et al. [19] further performed a security vulnerability assessment for a Transas Navi Sailor 4000 ECDIS installed on a Windows 7 operating system using a vulnerability scanning tool in the ECDIS software practice environment of a university. Vulnerable versions of SMB and Remote Desktop Protocol (RDP) used in the system were described as a critical vulnerability.

Svilicic et al. [20] performed a security vulnerability assessment using a scanning tool on the Navi Sailor 4000 ECDIS (Wärtsilä Transas, Finland), installed on the training/research ship *Kraljica Mora*. An outdated Apache web server and a vulnerable version of SMB were identified as vulnerabilities.

Finally, Svilicic et al. [21] performed a security vulnerability assessment using a scanning tool on the NACOS MULTIPILOT Platinum 2017 ECDIS system (Wärtsilä SAM Electronics GmbH, Germany), installed on a *ROPAX* vessel carrying passengers and cargo. These studies indicate the use of vulnerable versions of the SMB and RDP as a vulnerability.

Attack surfaces for the various systems of a ship were identified and analyzed by Hyra et al. [8], who described the risk levels of these attack surfaces using risk assessment methodologies to quantify asset importance, vulnerability importance, threat importance, and probability of occurrence.

Shang et al. [22] proposed a risk assessment method for ship control systems using fuzzy sets and attack trees, and found that there was a high probability of an attack exploiting a buffer overflow vulnerability and a remote mail attack. Furthermore, there was an attack path using an encryption cracking algorithm for onboard computer software.

In addition, Awan and Ghamdi [23] collected and analyzed cases of ship safety accidents that could affect cybersecurity. For instance, in the sinking of a coal carrier in 2004, the electronic navigational chart (ENC) was not up to date and failed to display the shallow water depth. This suggests that an inaccurate ENC file can lead to the sinking of a ship.

Caprolu et al. [24] discovered that ships using the NMEA-2000 standards were outfitted with various automation systems that were connected to the majority of the onboard equipment of the ships, and explained that the CAN-bus-based NMEA-2000 was vulnerable to attacks such as system shutdown and DoS attacks due to eavesdropping on unencrypted message contents and the injection of fake messages.

Common protocols were analyzed by Pavur et al. [25], who collected VSAT communication traffic used in marine satellite communication of ships. They found that marine VSATs were not encrypted and were vulnerable to eavesdropping and spoofing attacks because of the use of unencrypted protocols such as HTTP.

Tierney et al. [26] found a security vulnerability in Dualog, a software that provides services allowing the crew to connect to the Internet, use file transfers, and send emails on ships. These vulnerabilities included CVE-2020-26576, CVE-2020-26577, CVE-2020-26578, CVE-2020-26579, CVE-2020-26580, and CVE-2020-26581. In particular, it was found to be possible to increase the level of permissions by logging in with the default account (admin.g4@g4.dulog.no) and password.

Tierney et al. [27] boarded a large passenger ship and connected to the VSAT installed in the ship’s server room using the default password and the passenger’s Wi-Fi as a security test. They found that it was possible to access the cargo loading management system using MOXA IP-to-serial converters, as well as the closed-circuit television management system using the RTSP installed inside the ship. The most serious problem was that all information could be intercepted by physically sniffing and accessing the network switch and using the default password.

Dumbala [28] described ship operating technology (OT) systems as satellite communication and internal communication systems, propulsion, steering navigation and power control systems, cargo control rooms, and BWMSs. Threats and mitigation measures for the OT systems were described.

Jones et al. [29] proposed attack scenarios using system vulnerabilities, outdated software, and insecure network connections. Outdated software is used because many ships were built before cybersecurity became a concern. Older software is more prone to vulnerabilities, and some systems have been found to lack security patches.

Alcaide et al. [30] explained that a ship’s automatic identification system is one of the systems vulnerable to potential cyberattacks. By altering the actual position of a ship or injecting a false signal, it can affect the ship’s collision or operation suspension.

Finally, Haynes [31] reported on IRGC cyber operations documents, which included a plan to capsize or sink a ship by attacking the marine satellite communication terminal and the BWMS. The reviewed literature is summarized in Table 2.

### 3.1. MITRE ATT&CK-Based Ship Cyberattack Cases

We examined ship cyberthreats through a review of the literature. Based on this review, four cases with high impacts of cyberattacks on ships were selected. Case 1 is a ship sinking, Cases 2 and 3 are the wrong route, and Case 4 is an attack on the ship control system. MITER ATT&CK is described as TTPs (tactics, techniques, procedures). We analyzed the threats of the investigated ship systems, mapped them with techniques, and mapped the tactics and procedures connected to them. This explains the mitigation, as well. The four cases are as follows:

- Case 1: the IRGC plan to attack and sink ships;
- Case 2: cyberattacks on the INSs on ships, analyzed using the MITRE;
- Case 3: security vulnerabilities of the ECDIS of real training ships;
- Case 4: a threat analysis report of a ship’s OT system.

(1) Use Case 1: The IRGC BWMS Cyberattack Plans to Cause a Ship to Sink or Overturn

A cyberattack report created by the Intelligence Team 13 of the IRGC was revealed [10] that includes methods for hacking the BWMS on a ship to sink it. The BWMS system consists of a tank installed at the bottom of the ship as well as on the left and right sides of the hull to maintain the ship’s center of gravity when the ship is operating. A pump is used to fill or empty the tank with seawater. Under international law, its installation is mandatory to avoid environmental contamination, such as ecosystem disruption caused by the ballast water stored in the tank. The ship’s pumps are controlled by the Human Machine Interface (HMI) and can be monitored onshore through a satellite communication system. The report studied methods of intrusion from publicly available information.

There have been cases of similar attacks. In December 2020, an Iranian hacker group hacked Israel’s water facility industrial control system (ICS) and released a video that showed them accessing the HMI system. In this system, the Modbus/TCP port 502 was open to the Internet, and it was vulnerable to unauthorized access. The adversary was able to manipulate the water pressure and temperature [32]. This example supports the prediction of attacks on the BWMS of a ship.

A plan devised by a cyberattack force to hack a ship and inflict physical damage is threatening. A BWMS is generally installed on bulker and tanker ships. To prevent the ship from capsizing when it is tilted, the BWMS pump must operate with precision, and failure of this system may cause the ship to sink.

The attack begins by sending commands remotely from land to the ship via maritime satellite communications equipment. This attack would use a Seagull 5000i and Sealink CIR, which are marine satellite communication devices mounted on ships. Onboard and onshore connections have increased with the increasing need to access ships remotely.

The IRGC planned the attack in three phases. The first step is to determine the model of the marine satellite communication equipment that can access a ship at sea from the land through an IoT search engine. Thuraya and Wideye, which are manufacturers of marine satellite communication equipment in the UAE and Singapore, respectively, were mentioned. As of July 2021, 43 BWMS models have obtained type approval, and an adversary could select one of these models as a target. Because systems mounted on ships undergo mandatory approval, it is easy to obtain the manufacturer and model information. This step corresponds with *Reconnaissance*. The Thuraya devices mentioned in the report have hardcoded credentials vulnerabilities that have been identified in CVE-2013-6034 and CVE-2013-6035. Ship equipment is more difficult to update and patch than information technology equipment. Many ships still in service may have this vulnerability. This step corresponds with *Resource Development*. The adversary can gain access to the ship’s network through a known valid account, which can also be executed remotely, and corresponds with *Initial Access*. The next step involves modulating the data collected and processed by the BWMS system from the marine satellite communication terminal inside the hull. A PC is connected to the control and monitoring system that manages the BWMS on board. The BWMS of the internal network should be accessible from the marine satellite communication terminal, and hence the terminal and BWMS are connected by the same network. This step corresponds with *Lateral Movement*.

**Figure 2:**
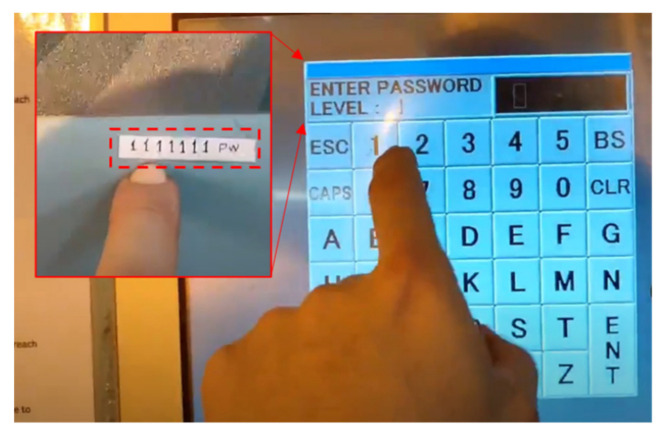
BWMS password exposure via SNS images of the crew.

During the analysis in this study, a new threat corresponding with the *Reconnaissance* phase was discovered. A scene showing the password (“1111111”) to the ship’s BWMS was uploaded by a crew member to a social network service and identified (Figure 2). This information is useful to an adversary. Other media uploaded by this crew member included information such as the name of the ship and onboard equipment, which could make it a target.

The first step would be to execute a sinking scenario, which requires identifying the attack vector for the attack surface of the BWMS. As mentioned in the report, in terms of cybersecurity, this is explained by the attack surface and vectors that affect the BWMS system and cause physical sinking. In terms of hardware, the attack interferes with the accurate operation of the pump and pressure gauge by inducing sensor malfunction or blocking/modulating sensor status information. This step corresponds with *Command and Control*.

In terms of software, the attack corrupts the management server file system or causes server errors. A file tampering attack is required if the pump management software is updated. This step corresponds with *Impact*.

**Figure 3:**
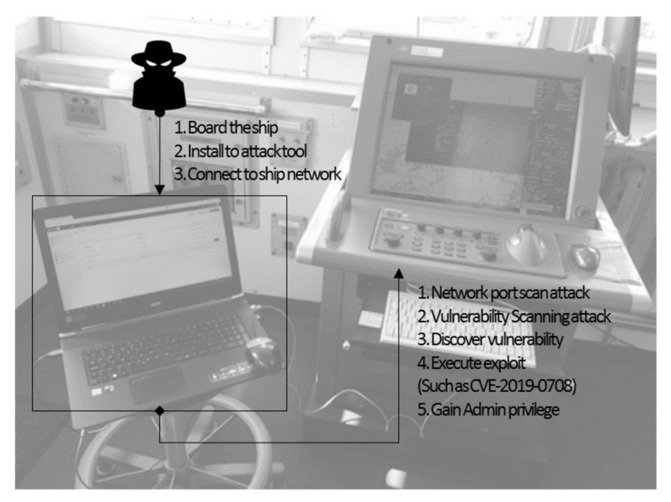
Scenario of attack case 1.

Table 3 shows an analysis of the plan devised by the Intelligence Team 13 in terms of an MITRE ATT&CK. Figure 3 shows the overall flow of the attack.

(2) Use Case 2: INS Attack [17]

The INS is a system that integrates and manages multiple devices connected with the ship radar, ECDIS (an electronic chart system), sonar, and GPS. Integrity among the systems is important for ships to operate on a normal route. A method was proposed for conducting an attack on the INS while the attacker is on board. System information is obtained by physically accessing a laptop with the ECDIS software installed, and the operating system information, the ship’s network IP, and the port number are identified. This step corresponds with the *Reconnaissance* phase. Its aim is to modulate the Winsock dynamic link library (DLL) file for an MiTM attack on the ECDIS software.

The target is an NMEA command. NMEA is a CAN-based protocol and has no safeguards, such as encryption. The protocol only involves a checksum, and the checksum can be easily bypassed. This step corresponds with the *Command* and *Control* phases. The adversary injects the payload stored on the USB into the ECDIS. This software has a built-in function to prevent keyboard inputs, allowing only mouse operations. However, the “Windows + R” keyboard shortcut can be used. An on-screen keyboard can be used for the keyboard input. It is possible to run commands on the operating system and have an administrator log into the ECDIS. This step corresponds with the *Defense Evasion* phase.

Some ECDISs use a factory default password, 0000. This step corresponds with the *Defense Evasion*, *Persistence*, *Privilege Escalation*, and *Initial Access* phases. A MITRE technique can correspond to multiple tactical phases. The adversary runs the payload from the USB. This step corresponds with the *Persistence* and *Privilege Escalation* phases. This tricks the ECDIS software into loading a fake Winsock DLL in a running operating system using administrator privileges. This step corresponds with the *Initial Access* phase. A fake Winsock DLL is then copied and installed. This step corresponds with the *Execution* phase.

The folder and registry of the ECDIS software are then updated. A fake Winsock DLL modulates the NMEA data or causes an ECDIS to “blue screen” when certain waters are reached. This scenario exhibits an air gap method that enables physical access, and the navigation system of the ship can be manipulated. It is possible to make the ship sail off course or go to the wrong destination by altering the NMEA text. Alternatively, the ship could move to shallow waters and be stranded. This step corresponds to the *Command* and *Control* phases.

**Figure 4:**
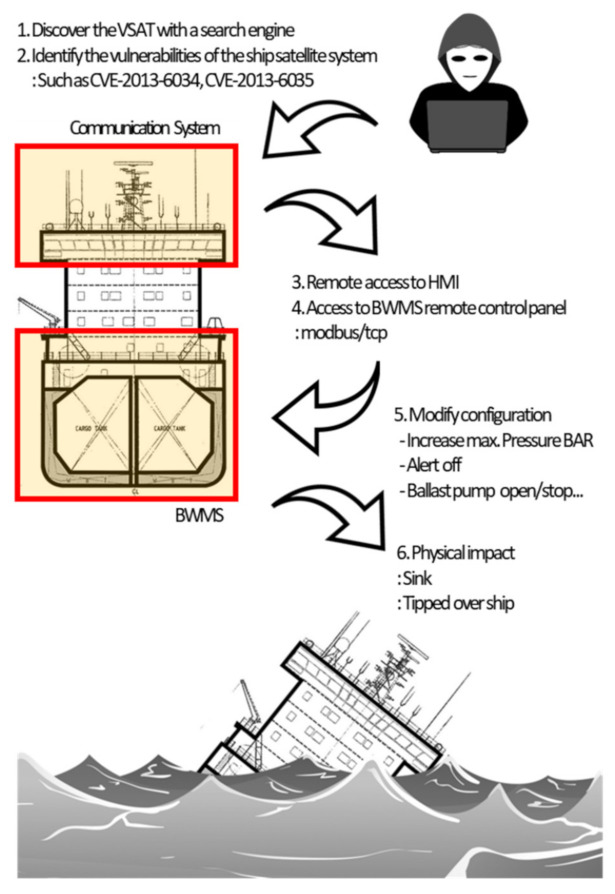
Scenario of attack case 2.

The scenario is mapped using the MITRE matrix in Table 3. This attack scenario was executed in August 2018 by installing the ECDIS software for testing on a real ship in offshore waters off the coast of Norway. The ECDIS needs to periodically update the ENC file. Although marine satellite communication may also be used, many ships are still updated using a CD, DVD, or USB. An update using a USB, CD, or DVD infected with malicious code, e.g., autorun.inf, can automatically execute the malicious code. The analysis of this cyberattack is listed in Table 4 in terms of an MITRE ATT&CK, and Figure 4 shows the overall flow of the attack.

(3) Use Case 3: ECDIS Attack [7]

**Figure 5:**
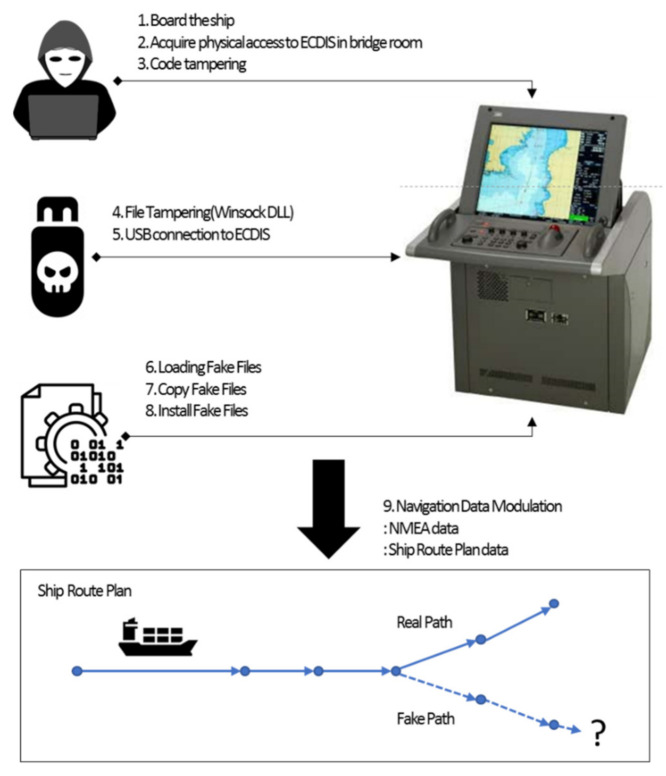
Scenario of attack case 3.

A security vulnerability scanner was used to conduct a cyberattack experiment on a real ship, a training ship from Kobe University, Japan. Figure 5 shows the overall flow of the experiment.

The aim of this experiment was to de Replaced image.termine security vulnerabilities. This allowed us to check the ship’s attack model. The cyberattack carried out by the researcher consisted of three steps. The first step was to board a real ship. Boarding a ship involves complex permits. This corresponds with the *Resource Development* phase. The adversary approaches the hull network to gain network access using a laptop. This corresponds with the *Initial Access* phase. After monitoring the ship’s network, the target ECDIS is identified. The adversary can obtain packets from the target ship’s network to inspect the entire packet or access the target through port scanning. This corresponds with the *Reconnaissance* phase.

The next step is to upload the security vulnerability scanning tool. This corresponds with the *Resource Development* phase. The tool can be installed via the onboard network or a USB device and corresponds with the *Initial Access* phase.

In the final step, the adversary performs vulnerability scanning on the real ship network, which is the *Execution* phase. The adversary identified a total of 37 security vulnerabilities, which is the *Discovery* phase. The ECDIS used Windows XP, which is an outdated operating system. The remote procedure call (RPC) and SMB with remote code execution vulnerability were used. The RPC has a remote code execution vulnerability that can be exploited, and there are many attack codes for the RPC, SMB, and RDP of outdated operating systems. For instance, an adversary could use CVE-2019-0708. The service pack was not installed on the operating system, Windows XP Embedded, where the ECDIS software was installed. This corresponds with the *Lateral Movement* phase.

An analysis of the cyberattack is listed in Table 5 in terms of the MITRE ATT&CK. Figure 5 shows the overall flow of the attack.

(4) Use Case 4: Ship Operating Technology (OT) System Threat Analysis [27]

The ship OT system has been classified, as shown in Table 6, and attackable threats have been defined. The analysis was conducted using the attack techniques and tactics of MITRE ATT&CK for ICSs, which were created to analyze the OT environment.

Table 7 describes the results of the analysis by the MITRE ATT&CK for ICS for the communication system.

Table 8 shows the results of the analysis by the MITRE ATT&CK for ICS for the propulsion, machinery, and power control systems.

Finally, Table 9 shows the results of the analysis by the MITRE ATT&CK for ICS for the navigation system.

**Figure 6:**
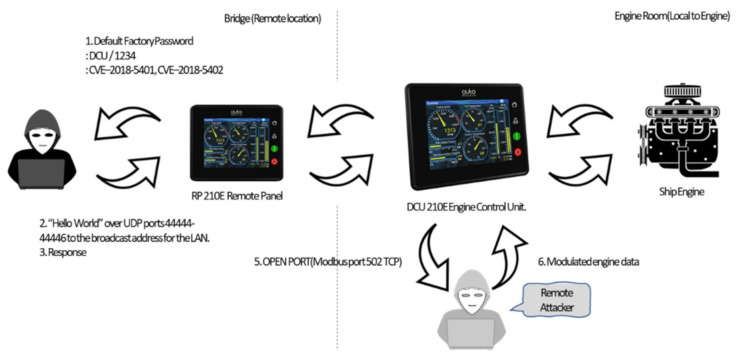
Attack scenario using CVE.

A ship consists of various OT systems. Possible attack scenarios in the ship OT environment can be explained through the CVE of the ship engine control system. Figure 6 shows the overall flow of the scenario.

Auto-Maskin remote panels (RPs) and DCU control units were used to control and monitor ship engines. The adversary can attempt to use the factory default password (DCU/1234) from the manual of the RP 210E product installed on the bridge or use CVE–2018-5401 and CVE-2018-5402 to gain access without permission. In DCU 210E, CVE-2018-5400 can be used for spoofing or relay attacks with Modbus-TCP. Arbitrary messages can be sent to DCU or RP devices remotely without entering the engine room.

### 3.2. TTPs Analysis

**Figure 7:**
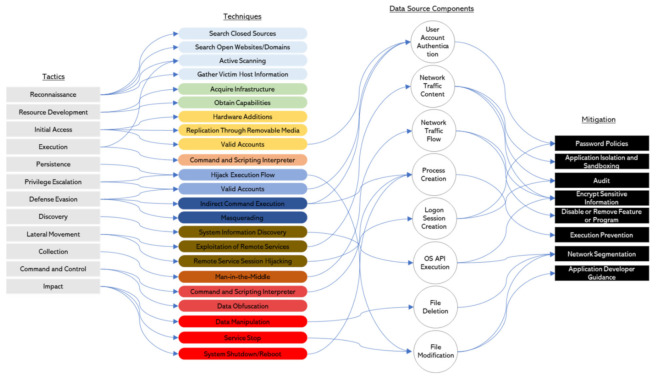
Analysis of ship TTPs by MITRE ATT&CK Enterprise.

**Figure 8:**
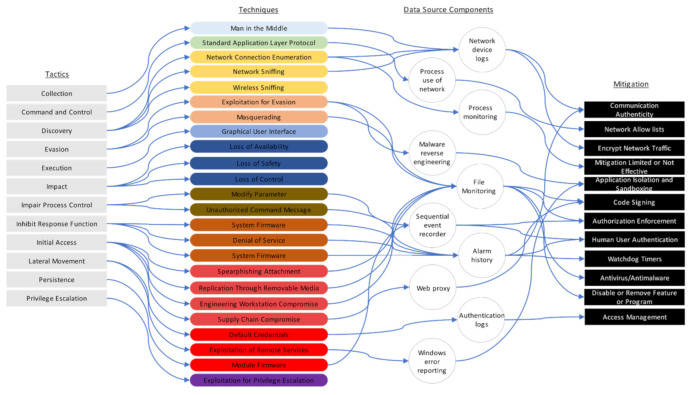
Analysis of ship TTPs by MITRE ATT&CK ICS.

The use of the MITRE ATT&CK framework to analyze cyberattacks on ships allows the use of the behavior matrix to plan and design countermeasures. Effective countermeasures can be mapped based on the important cybersecurity cases of the ships analyzed in this study. The list of countermeasures is not complete, and it is not necessary to apply all of the mitigation measures listed. Different measures may be required depending on the ship type and system. More robust countermeasures are required for warships. This study considers cases 1, 2, and 3 (analyzed by MITRE ATT&CK Enterprise) and case 4 (analyzed by MITRE ATT&CK ICS by TTPs), and suggests mitigation plans for them. These are shown in Figure 7 and Figure 8. The techniques reveal the patterns in which adversarial behavior occurs. The data source components explain the intervals in which the patterns occur.

(1) MITRE ATT&CK Enterprise

An explanation of Figure 7 follows. Eight mitigation plans are suggested using 12 tactics, 23 techniques, and 8 data source components. More diverse mitigation measures can be suggested. An analysis was conducted based on the threats identified in these cases. The data source components refer to attack behaviors. The *Reconnaissance* and *Resource Development* tactics occur before the adversary physically approaches the ship. It is difficult to effectively defend these tactics. Efforts to minimize the attack surfaces of the target ship, supply chain, and crew are required. Network segmentation of the onboard system is difficult when underway. Hence, mitigation plans should consider the conditions of the ship and the operating environment.

(2) MITRE ATT&CK ICS

An explanation of Figure 8 follows. Twelve mitigation plans are suggested using 12 tactics, 23 techniques, and 10 data source components. An attack on the ship control system can be detected in the alarm history of network equipment log and file monitoring. However, device-based mitigation measures are difficult to modify firmware.

## 4. Considerations and Limitations

Modern ICT-based ships are highly vulnerable to cyberattacks. If the system or navigation equipment of a ship is compromised, an attacker can gain control of the ship and sink it. The analysis of the attack model is the initial step toward understanding ship cybersecurity; furthermore, it allows appropriate security requirements to be established. The MITRE ATT&CK framework classifies attack behavior into a detailed knowledge base. Herein, mitigation measures were presented by classifying the ship attack model as a knowledge base. Ships required both MITRE ATT&CK Enterprise and the ICS framework, as IT/OT is used together. Attack technology is evolving. The proper use of these two frameworks and continuous hardening are required.

## 5. Conclusions

For the safe navigation of ships, cybersecurity regulations enforced by the IMO as well as the guidelines of governments (e.g., USCG) and related associations (e.g., BIMCO) should be observed. Shipbuilders and ship equipment manufacturers also need to understand the threats in threat libraries and implement appropriate measures.

This study analyzed ship cybersecurity research cases based on the MITRE ATT&CK framework and presented mitigation plans based on the analysis results.

In this study, we investigated and analyzed potential cybersecurity threats on ships. It was established that while ICT-based ships are vulnerable to sophisticated cyberattacks, the industry is not yet fully prepared. Future research on ship cybersecurity is required, and future research trends are as follows:

1. Design of cybersecurity risk management and risk assessment framework for various ship types [33].
2. Threat analysis and security requirements analysis for NMEA 0183, 2000 protocol [34].
3. A honeypot system to detect new cyberattacks onboard and reduce false positives in IDS [35]. In particular, a honeypot system has been developed [36] that can be installed in an L2 layer switch environment using VLAN when the deployment location of the honeypot is the ship’s internal network. For analyzing the attack type on the ship’s satellite communication system from outside the ship, a cloud-based ICS/SCADA honeypot system has been proposed [37].
4. Artificial intelligence-based ship cyber threat response technology for an independent system of an autonomous vessel at sea to recognize an attack and respond by itself.
5. The ship equipment whitelisting technique and data set [38].
6. Maritime cyberthreat intelligence: technology for the classification and evaluation of threat information tailored to ships, the maritime industry, and stakeholders [39].
7. Ship digital forensic technology to collect and analyze digital evidence in security incidents of onboard systems.

Cybersecurity is an important issue for ICT-based ships. There is an urgent need to increase awareness regarding the various threats posed to systems such as ECDIS, VDR, ECS, VSAT, and NMEA, as various vulnerabilities are being discovered in IT and OT every day. From a cybersecurity perspective, this awareness is critical to ensure the dependability of a ship at sea. We believe that our research will instigate future research directions and provide motivation for further research on ship cybersecurity.

## Figures and Tables

**Table 1:** MITRE ATT&CK tactics.

Tactic	Description
*Reconnaissance*	The adversary attempts to gather information they can use to plan future operations.
*Resource Development*	The adversary attempts to establish resources they can use to support operations.
*Initial Access*	The adversary attempts to gain access to the target network.
*Execution*	The adversary attempts to run malicious code.
*Persistence*	The adversary attempts to maintain their progress.
*Privilege Escalation*	The adversary attempts to obtain higher-level permissions.
*Defense Evasion*	The adversary attempts to avoid detection.
*Credential Access*	The adversary attempts to steal account names and passwords.
*Discovery*	The adversary attempts to determine the target environment.
*Lateral Movement*	The adversary attempts to move through the target environment.
*Collection*	The adversary attempts to gather data of interest to their goal.
*Command and Control*	The adversary attempts to communicate with compromised systems to control them.
*Exfiltration*	The adversary attempts to steal data.
*Impact*	The adversary attempts to manipulate, interrupt, or destroy the target systems and data.

**Table 2:** Summary of research.

System	Threat	Impact	Reference
AIS	AIS malfunction, jamming, spoofing	Not mentioned	Kessler et al. [16]
	Spoofing, replay attack, frequency hopping attack	Not mentioned	Dumbala [28]
	Designed without security, malicious version, malware	Hijacking, smuggling, theft	Jones, K.D. et al. [29]
	False signals, represent nonexistent emergencies	Collisions, pollution, grounding, interruption of port operations	Alcaide and Llave [30]
ECDIS	Physical access Internet connection establishment Authorized access Operating system support and security patches Operating system configuration etc.	Provides physical/logical access Exploitation of well-known vulnerabilities Reduces performance and opens backdoor	Svilicic et al. [7]
	Vulnerable versions of SMB and Remote Desktop Protocol (RDP)	Infection and dysfunctionality of all ECDIS stations in the network	Svilicic et al. [19]
	Outdated Apache web server Vulnerable version of SMB	Gain unauthorized access Remote attacker	Svilicic et al. [20]
	Vulnerable versions of SMB and Remote Desktop Protocol (RDP)	Execute arbitrary code without authentication Disclose sensitive information	Svilicic et al. [21]
	Designed without security, malicious version, malware	Hijacking, smuggling, theft	Jones, K.D. et al. [29]
	GNS spoofing by malware	Sails to different coordinates Crash the operator station	Lund et al. [18]
	Virus, DoS, spoofing	Not mentioned	Dumbala [28]
SATCOM	Backdoors Hardcoded credentials Insecure protocols Undocumented protocols Password reset weaknesses	Install malicious firmware Execute arbitrary code	Santamarta [4]
	Unencrypted protocols	Disclose sensitive information	Pavur et al. [25]
	Default credentials, not updated software, etc.	Not mentioned	Dumbala [28]
	Cyberattack by hostile states	Disclose sensitive information	Haynes [31]
BWMS	Phishing emails, malware	Ransomware, false command	Dumbala [28]
	Cyberattack by hostile states	Sinking of the ship	Haynes [31]
	Designed without security, malicious version, malware	Hijacking, smuggling, theft	Jones, K.D et al. [29]
other	NMEA: unencrypted message DoS attacks	Injection of fake messages System shutdown	Caprolu et al. [24]
	Inaccurate ENC file	Sinking of the ship	Awan and Ghamdi [23]
	Default account and password	Elevation privilege	Tierney et al. [26]
	Default password and the passenger’s Wi-Fi	Access system inside the ship Information intercept	Tierney et al. [27]
	Described the risk levels of these attack surfaces using risk assessment methodologies		Hyra et al. [8]
	Proposed a risk assessment method for ship control systems using fuzzy sets and attack trees		Shang et al. [22]

**Table 3:** MITRE ATT&CK analysis results for attack case 1.

Tactic	Description
*Reconnaissance*	The adversary obtains information on the equipment of the target ship. The adversary searches for marine satellite communication equipment. Attackers can utilize IoT search engines. Examples of search keyword in censys: services.banner = “sailor 600” The adversary obtains the BWMS password through the Social Network Service of the crew member (Figure 3).
*Resource Development*	The adversary identifies the known vulnerabilities of maritime satellite communication terminals (CVE-2013-6034, CVE-2013-6035). Some satellite communication devices can be accessed using TCP port 1827 without authentication. The adversary identifies a valid account at the maritime satellite communication terminal. Some devices use the default account (“admin”).
*Initial Access*	The adversary accesses the onboard network with a valid account.
*Lateral Movement*	The adversary accesses the BWMS via the onboard network.
*Command and Control*	The adversary attacks the BWMS attack surfaces. The adversary executes the BWMS attack vectors. The adversary modulates the pump data (running the port side pump or the starboard side pump). The adversary turns off the pump alarm.
*Impact*	The ship sinks. The ship capsizes.

**Table 4:** MITRE ATT&CK analysis results for attack case 2.

Tactic	Description
*Reconnaissance*	The adversary acquires ship boarding rights. The adversary physically accesses the ECDIS software.
*Initial Access*	The adversary uses the ECDIS factory default password (pw: 0000) to gain access.
*Defense Evasion*	A fake Windock DLL is loaded. : The procedure for performing a DLL injection attack is as follows.(1) Attach the process—(2) Allocate memory within the process—(3) Copy the DLL or DLL path to the process memory and determine the appropriate memory address—(4) Instruct the process to run the fake DLL. The adversary injects the payload stored in the USB into the ECDIS. The adversary activates the keyboard with the “Windows + R” keyboard shortcut.
*Execution*	A fake Winsock DLL is copied and installed.
*Persistence*	The adversary executes the payload.
*Collection*	The adversary modifies the Winsock DLL file for an MiTM attack by the ECDIS software.
*Command and Control*	The ship goes off course because the NMEA command is altered.
*Impact*	The ECDIS is paralyzed (blue screen). The ship is stranded. The ship goes off course.

**Table 5:** MITRE ATT&CK analysis results attack case 3.

Tactic	Description
*Resource Development*	The adversary boards the ship. The adversary uploads the security vulnerability scanning tool. The adversary scans network ports.
*Initial Access*	The adversary installs the security vulnerability scanning tool. The adversary performs installation via onboard network or USB devices.
*Reconnaissance*	The adversary identifies the target ECDIS through port scanning.
*Execution*	Network vulnerability scanning on ship networks is performed.
*Discovery*	The adversary identifies a total of 37 security vulnerabilities.
*Lateral Movement*	The adversary runs the exploit code to attack the venerability (CVE-2019-0708). : This vulnerability is called the BlueKeep vulnerability. This means that if an unauthenticated attacker connects to the target system using the Remote Desktop Protocol (RDP) and sends an attack request, the code will be executed without permission.
*Impact*	The adversary manipulates the ECDIS system operation data.

**Table 6:** Ship OT system types and their components.

Type	System
*Communication Systems*	Satellite communication system Integrated communication system Voice over internet protocol Wireless LAN
*Propulsion, Machinery and Power Control Systems*	Engine governor system Fuel oil system Alarm monitoring and control system Power management system Emergency generator and batteries
*Navigation Systems*	ECDIS RADAR AIS GPS Dynamic Positioning System Global Maritime Distress and Safety System VDR
*Cargo Management System*	Cargo control room Ballast water system

**Table 7:** Communication system analysis results.

Tactic	Description
*Command and Control*	The use of a weak protocol is susceptible to data falsification. The adversary attacks vulnerable protocols such as HTTP and TELNET.
*Lateral Movement*	The adversary takes advantage of the default settings and passwords set by the manufacturer on the ICS system device. The factory default password is written in the manual.
*Execution*	The adversary attacks the web interface used on the ship.
*Initial Access*	The adversary executes targeted phishing attacks on crew members using malware.
*Discovery*	Network eavesdropping on Voice over Internet Protocol (VoIP) can expose sensitive information.
*Inhibit Response Function*	It is difficult to rapidly update software for OT systems. The denial-of-service attack on VoIP disrupts hull communication.
*Impact*	Communication failure occurs. The ship collides.

**Table 8:** Propulsion, machinery, and power control system analysis results.

Tactic	Description
*Initial Access*	The adversary stores malware on removable media. OT environments such as propulsion and control systems are located at the bottom of the ship. Access permission is required. The adversary inserts removable media into the OT system.
*Collection*	The adversary with access to the ship’s onboard network can use the MiTM attack to modulate network traffic in real time. The ship OT system uses NMEA2K.
*Impact*	The ship is stranded. The ship collides.

**Table 9:** Navigation system analysis results.

Tactic	Description
*Initial Access*	The adversary inserts removable media into the onboard system. The adversary sends spear phishing emails to crew members’ emails. The adversary approaches using the trusted supply chain.
*Persistence*	The adversary identifies the vulnerabilities in ship OT systems. The adversary tampers with firmware.
*Privilege Escalation*	A crew member receives an email and downloads the attachment. The attack code stored in the removable media is executed.
*Evasion*	NMEA packet is modulated (direction, latitude, longitude, velocity, and depth). OT data are modulated (gauge information).
*Discovery*	The adversary eavesdrops on the Wi-Fi network.
*Lateral Movement*	The adversary sends the attack code to the attack surface of the ship.
*Inhibit Response Function*	The adversary executes a DoS attack on the ship networks.
*Impair Process Control*	The adversary modifies the system and sensor parameters. The adversary sends a message of system outage.
*Impact*	The ship is stranded. The ship collides.

## Data Availability

Not applicable.
